# Cobalt-Based Metal-Organic Frameworks and Their Derivatives for Hydrogen Evolution Reaction

**DOI:** 10.3389/fchem.2020.592915

**Published:** 2020-11-20

**Authors:** Wenjuan Han, Minhan Li, Yuanyuan Ma, Jianping Yang

**Affiliations:** State Key Laboratory for Modification of Chemical Fibers and Polymer Materials, College of Materials Science and Engineering, Donghua University, Shanghai, China

**Keywords:** electrocatalysts, metal-organic frameworks, cobalt-base catalysts, hydrogen evolution reaction, water electrolysis

## Abstract

Hydrogen has been considered as a promising alternative energy to replace fossil fuels. Electrochemical water splitting, as a green and renewable method for hydrogen production, has been drawing more and more attention. In order to improve hydrogen production efficiency and lower energy consumption, efficient catalysts are required to drive the hydrogen evolution reaction (HER). Cobalt (Co)-based metal-organic frameworks (MOFs) are porous materials with tunable structure, adjustable pores and large specific surface areas, which has attracted great attention in the field of electrocatalysis. In this review, we focus on the recent progress of Co-based metal-organic frameworks and their derivatives, including their compositions, morphologies, architectures and electrochemical performances. The challenges and development prospects related to Co-based metal-organic frameworks as HER electrocatalysts are also discussed, which might provide some insight in electrochemical water splitting for future development.

**Graphical Abstract:**
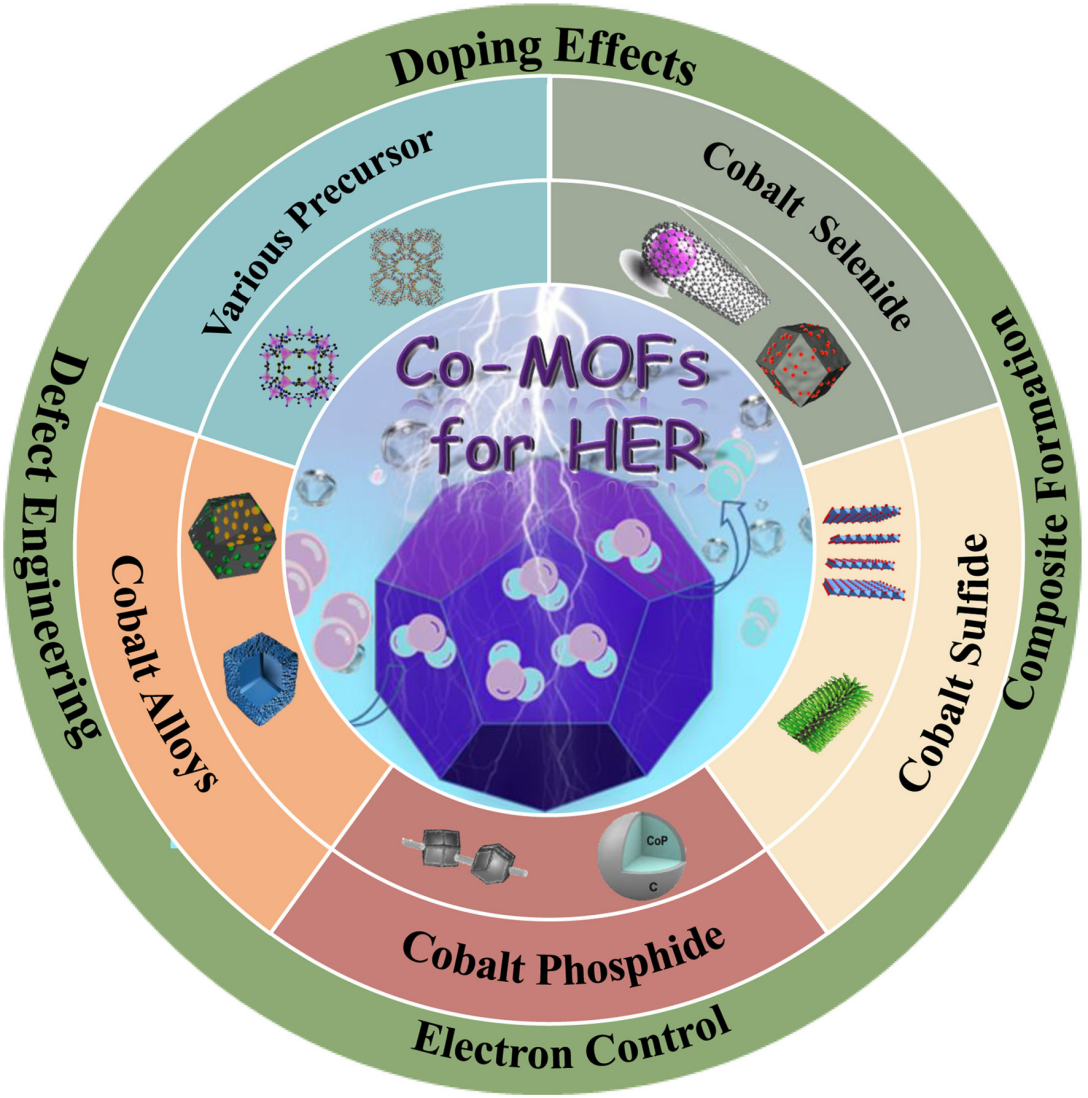


## Introduction

With the rapid development of economy, energy and environmental problems have raised increasing concerns in recent years (Su et al., 2019; Lin et al., 2020; Liu H. et al., 2020; Liu S. et al., 2020b). To reduce the fossil fuels reliance and lower greenhouse gas emission, there is an urgent need to develop clean and sustainable energy resources. Hydrogen, which possesses high gravimetric energy density, has been considered as an ideal alternative energy carrier to fossil fuels (He et al., 2020). The green and sustainable supply of hydrogen is essential for the hydrogen economy. At present, hydrogen is mainly obtained through a steam reforming of fossil fuels, which not only consumes a large amount of non-renewable energy, but also increases CO_2_ emissions (Qin et al., 2016; Li M. et al., 2017a,b). Therefore, to produce hydrogen in a clean and renewable way is urgently required. Water electrolysis, featuring high energy conversion efficiency, high hydrogen production rate and compact devices, has been regarded as an ideal method for hydrogen production in the future (He et al., 2020).

**Figure 1:**
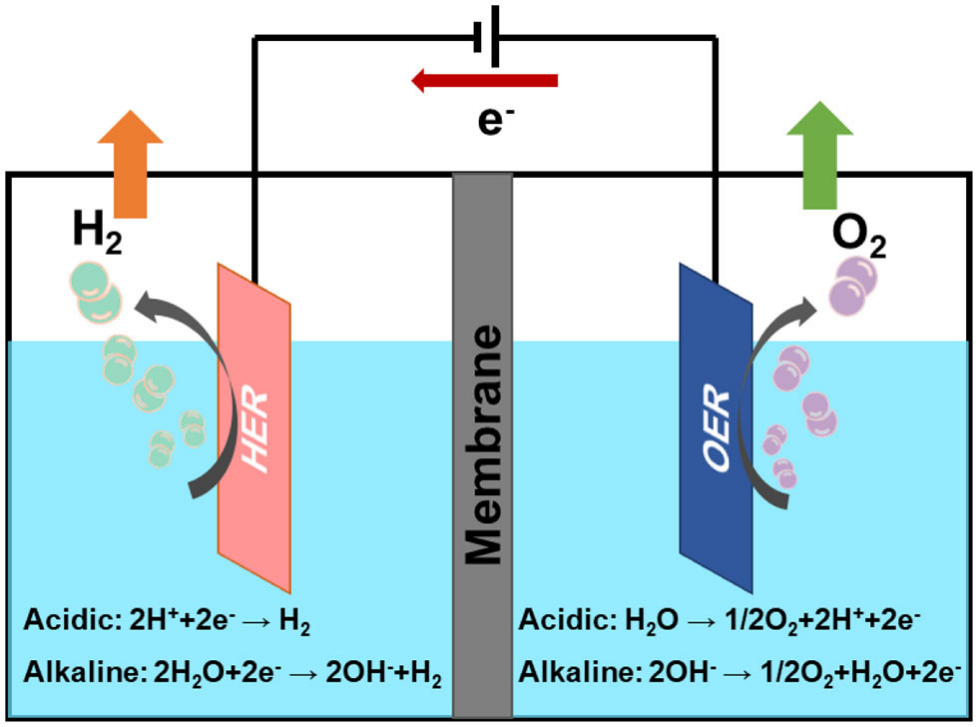
Schematic of water electrolysis system.

The electrochemical water splitting is composed of two half reactions (Figure 1): HER on cathode and oxygen evolution reaction (OER) on anode. Two electrodes of the electrolysis system will play a key role in the hydrogen production. In theory, the decomposition voltage of water is 1.23 V. However, in order to overcome the thermodynamic equilibrium potential, a certain overpotential (η) is required during the practical electrolytic process, which will increase energy consumption (Xiang et al., 2020). Generally, highly efficient electrocatalysts could reduce the overpotential and increase the current density of these catalytic reactions (Karmodak and Andreussi, 2020; Xu et al., 2020). It is well-known that platinum group metals are the most efficient catalysts for HER (Tian et al., 2019; Huang C. et al., 2020; Huang H. et al., 2020b; Lan et al., 2020; Liu Z. et al., 2020). However, the scarcity and high cost of these precious metals impede their large-scale applications. Therefore, it is pressingly needed to develop low platinum or non-precious metal electrocatalysts with high catalytic activity and long cycle stability for hydrogen production, which will facilitate the realization of hydrogen economy.

During the past few years, a variety of electrocatalysts have been studied for HER, mainly including metals (Xiu et al., 2020), metal sulfides (Huang H. et al., 2020b), metal phosphides (Zhou et al., 2020), metal carbides (Ma X. C. et al., 2020; Yu H. et al., 2020; Yu Y. et al., 2020) and carbon-based materials (Wang H.-F. et al., 2020; Wang J. et al., 2020; Wang X. et al., 2020). In general, the electrocatalysis properties could be improved through increasing the number of active sites and the intrinsic activity of each active site (Huang H. et al., 2020b; Huang Z. et al., 2020). It is worth noting that the concentration and intrinsic activity of active sites can be simultaneously improved by increasing the specific surface area of catalyst (Eiler et al., 2020). In addition, regulating the electronic structure of catalysts could also increase the intrinsic activity, such as heteroatom doping (Liu et al., 2015; Nan et al., 2019; Liu H. et al., 2020), defect engineering (Yilmaz et al., 2018), alloying (Yang et al., 2015). Co is plentiful and low cost compared with noble metals. It also has high theoretical catalytic activity due to Co have a low energy barrier for H adsorption, making Co-based composites to be excellent candidates for HER (Chai et al., 2020). Therefore, Co-based catalysts have been widely investigated as the catalysts for HER (Wang H. et al., 2018; Wang X. et al., 2018a; Kuznetsov et al., 2019). Metal-organic frameworks are a porous material consisted of metal nodes and organic linkers, and their derived composites exhibit tunable structure, adjustable pores and large specific surface area (Huang H. et al., 2020a,b). Moreover, the high specific surface can provide a huge number of active sites and the open pore structure in the catalytic process, which is very important to increase the catalytic activity. Therefore, a large number of MOFs have been exploited and classified according to their structural characteristics in recent years, such as zeolitic imidazolate frameworks (ZIFs) (Liu H. et al., 2020), boron imidazolate frameworks (BIFs) (Liu et al., 2018), materials of institute lavoisier (MIL) (Chen J. et al., 2019; Chen W. et al., 2019) and so on. Through the pyrolysis process, these materials can convert into various metals, metal sulfides/phosphides/carbides, carbon-based materials, and other metal structures. Co-MOFs, showing excellent performance for the HER, has sprung up due to its intriguing advantages: (1) Co metal has a proper binding energy for hydrogen atom (close to Pt) (Jin et al., 2015; Huang et al., 2017); (2) the porosity of MOFs can improve the exposure of active sites and electron/mass transfer (Jia et al., 2017; Wang X. et al., 2020); (3) the organic linkers can serve as the source of N-doping which facilitates to maximize conductivity of carbon matrix (Li D. et al., 2018; Weng et al., 2018). In light of the above unique characteristics, Co-based MOFs have attracted great attention for obtaining highly efficient catalysts for HER.

In this review, we present an overview of Co-based MOFs for HER in the past few years. Firstly, the reaction mechanisms of hydrogen evolution reaction were briefly summarized, and also giving the design strategy of Co-based MOFs electrocatalysts. According to the related research works, we discussed the current progress of Co-based MOFs electrocatalysts. In addition, the challenges and perspectives for Co-based MOFs HER catalysts were also discussed, which might provide some insight in electrochemical water splitting for future development.

## Reaction Mechanism for HER

The reaction mechanism of HER has been extensively studied (Chen et al., 2020). It is generally carried out in acidic condition or alkaline condition. HER is a two-electron transfer process, includes three possible reaction steps. The specific reaction steps are as follows:

1. Volmer reaction:
  (1) $$\begin{array}{l} {\text{H}}_{3}{\text{O}}^{+}+\text{M}+{\text{e}}^{-}↔\text{M}{-}^{*}\text{H}+{\text{H}}_{2}\text{O }\left(\text{acidic medium}\right) \end{array}$$
  (2) $$\begin{array}{l} {\text{H}}_{2}\text{O}+\text{M}+{\text{e}}^{-}↔\text{M}{-}^{*}\text{H}+\text{O}{\text{H}}^{-}\left(\text{alkaline medium}\right) \end{array}$$
2. Heyrovsky reaction:
  (3) $$\begin{array}{l} {\text{H}}^{+}+{\text{e}}^{-}+\text{M}{-}^{*}\text{H}↔{\text{H}}_{2}+\text{M }\left(\text{acidicmedium}\right) \end{array}$$
  (4) $$\begin{array}{l} {\text{H}}_{2}\text{O}+{\text{e}}^{-}+\text{M}{-}^{*}\text{H}↔{\text{H}}_{2}+\text{O}{\text{H}}^{-}\\ +\text{ M}\left(\text{alkaline medium}\right) \end{array}$$
3. Tafel reaction:
  (5) $$\begin{array}{l} 2\text{M}{-}^{*}\text{H}↔{\text{H}}_{2}+2\text{M }\left(\text{acidic and alkaline medium}\right) \end{array}$$

**Figure 2:**
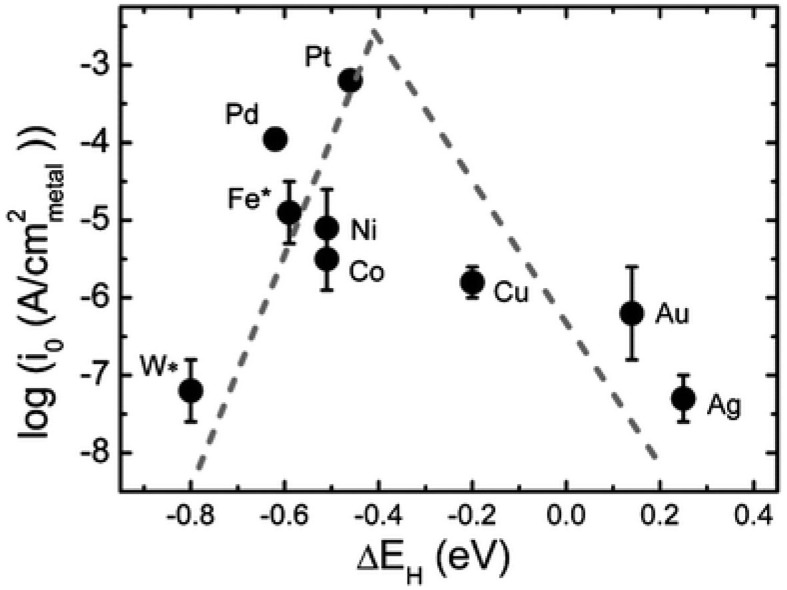
Volcano plot for HER in alkaline medium for various metals. Reproduced with permission (Sheng et al., 2013). Copyright © 2013, Energy Environ. Sci. All rights reserved.

M represents the hydrogen adsorption sites, ^*^H represents the reaction intermediates of hydrogen atom on catalyst. From the above reaction steps, the reaction mechanism of HER in acidic electrolyte and alkaline electrolyte is much more different. In acidic electrolyte, the formation of ^*^H is come from hydronium-ion (H_3_O^+^) during Volmer process, while in alkaline electrolyte ^*^H is formed by the dissociation of water molecules (H_2_O). Subsequently, the adsorbed ^*^H will react with H^+^ or H_2_O to produce H_2_ via Heyrovsky process, or combined with another ^*^H to generate H_2_ through Tafel process. The whole process of HER includes the ^*^H adsorption and hydrogen desorption from the active sites on the surface of electrocatalysts. Generally, the rate-determining step of HER is the adsorption free energy of hydrogen (Δ${\text{G}}_{{\text{H}}^{*}}$) (Nørskov et al., 2005). For excellent HER electrocatalysts, the bonding strength of the adsorbed hydrogen atom with catalyst should be appropriate. In alkaline media, however, it will introduce an additional energy barrier due to the dissociation of water molecule, which may lower the reaction rate of alkaline HER. It is obvious that the hydrogen adsorption and dissociation on the electrode surface are two consecutive steps in the electrocatalysis process (Li et al., 2020). However, they are inherently competitive. If the bonding strength between the catalyst and hydrogen atom is too weakly, it cannot effectively adsorb hydrogen proton intermediates. On the contrary, if the bonding strength between the catalyst and hydrogen atom is too strongly, the generated hydrogen is difficult to desorb from the catalyst. Therefore, only when the adsorption and desorption reach a balance, the HER performance can achieve the most excellent (Hossain et al., 2019). Skúlason et al. (2010) calculated the free energy of hydrogen adsorption of different transition metals using density functional theory, the results are consistent with the Sabatier principle. Sheng has summarized the volcano plots of various metals under alkaline conditions for the HER, which is shown in Figure 2 (Sheng et al., 2013). This volcano plot is a useful descriptor of hydrogen evolution activity for various metals. When the position of metal is close to the apex of volcano chart, the catalyst reaches the best balance of adsorption and desorption of hydrogen, which has a best HER performance.

According to the volcano plot, Co has a lower adsorption free energy of hydrogen, which should have excellent HER performance. However, there is still a certain gap between Co and precious metals, i.e., Pt, Pd. Hence, to improve the HER performance of Co-based catalysts are necessary. In view of the intrinsic features of large pore volumes, high specific surface area, tunable chemical constituents, and adjustable crystalline porous frameworks, Co-based MOFs have become an increasingly important catalysts in the field of HER. However, insufficient electrical conductivity and low chemical stability seriously limit their applications. Thus, to improve the HER performance of Co-based MOFs catalysts are of great importance. In recent years, various strategies have been adopted to improve the HER performance, including adjusting precursors and synthetic methods, doping heteroatoms, and alloying. Therefore, a reasonable design of Co-based MOFs catalysts can be an effective method to improve the electrocatalytic performance.

## Co-based MOFs Materials for HER

Co-based MOFs has been proved to have a low energy barrier for HER (Jin et al., 2015). However, it still has much room for improvement. Up to now, various strategies have been adopted to improve its electrocatalytic performance, such as choosing different precursor, selenizing, vulcanizing, phosphating, and alloying.

### Co-MOFs Derivatives Catalysts From Various Precursor

MOFs and MOFs-derived materials are excellent catalytic materials (Pan et al., 2018; Ma X. C. et al., 2020; Ma Y. et al., 2020). They have attracted much attention because of their designability and adjustability. The structure of MOFs depending on the bridging metal ions and organic linkers. In the past several years, various Co-MOFs derived materials have been extensively developed and used as electrocatalysts for HER (Huang et al., 2017; Li M. et al., 2017b; Tabassum et al., 2017).

With the development of B-H key function research (Zhang et al., 2014), BIFs are widely used in HER. There are two types of tetrahedral centers in BIFs: B (boron) and M (metal) (Zhang H. X. et al., 2016b; Zhang X. et al., 2016). BIFs are generally using lightweight main group metals to build the vertices of the framework and using light elements B to construct polyhedral nodes (Zhang et al., 2009; Zhang H. X. et al., 2016a; Zhang X. et al., 2016; Zhu et al., 2020). Liu et al. (2018) prepared Co/NBC by carbonization of a cobalt-based boron imidazolate frameworks (BIF-82-Co) under various pyrolysis temperature. In 1.0 M KOH solution, Co/NBC-900 required a lower overpotential of 117 mV to achieve the current density of 10 mA cm^−2^ for HER, and a small Tafel slope of 146 mV dec^−1^. However, the poor stability (10 h) of these catalysts seriously limits their practical applications.

ZIFs, which is based on imidazolate complexes, can serve as an excellent precursor for non-precious Co-MOFs catalysts. Yang et al. (2017) proposed CoP nanoparticles encapsulated in ultrathin nitrogen-doped porous carbon (CoP@NC) with ZIF-9 as the precursor. The CoP@NC catalyst exhibits outstanding HER catalytic activities in alkaline and acidic conditions. Remarkably, the CoP@NC achieves a current density of 10 mA cm^−2^ at an extremely low overpotentials of 129 and 78 mV in 1.0 M KOH and 0.5 M H_2_SO_4_ solutions, respectively. More recently, Sun et al. (2016) prepared the CoSe_2_/CF from zeolitic imidazolate framework-67 (ZIF-67) through pyrolysis and selenizing process. CoSe_2_/CF delivers a smaller Tafel slope of ~52 mV dec^−1^. In addition, CoSe_2_/CF also shows a better long-term stability than commercial Pt/C.

**Figure 3:**
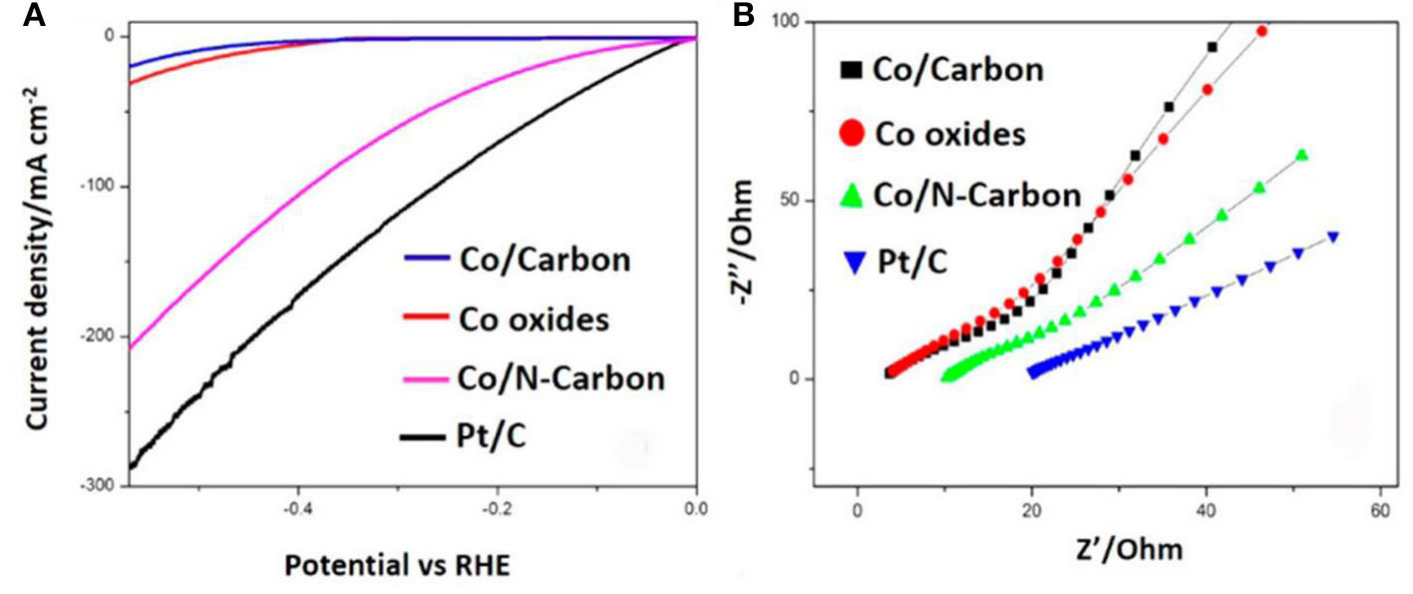
N-Doped carbon material derived Co/N-Carbon as an electrocatalyst for HER in 1 M KOH solution. **(A)** LSV of different electrocatalysts with rotation disk electrode at 1,600 rpm. **(B)** EIS of different electrocatalysts. Reproduced with permission (Huang et al., 2017). © 2017 ACS Sustain. Chem. Eng. All rights reserved.

Apart from MOFs precursors, carbon matrix also plays a decisive role of Co-MOFs derived HER catalysts. To date, most MOF-derived catalysts are modified via the heteroatom doping strategy. When the electronegativity of doping atoms is larger than that of carbon atoms, they will act as electron acceptors (i.e., N, O), on the contrary, they are called electron donors (i.e., F, S, P, B) (Zhang K. et al., 2017; Zhang L. et al., 2017). Nitrogen atom has a similar atomic size but one more shell electron compared with carbon. Therefore, N is the most common doping element among the above elements. The N doping can promote the electrocatalytic activity by increasing the conductivity, enhancing the adsorption strength of anion group (-OH), reducing the reaction energy barrier and accelerating the reaction kinetics (Li D. et al., 2018). Generally, there are two ways to introduce nitrogen atom: one is to select a precursor containing N elements, the other is to combine MOFs with N-containing materials, then allowing the heteroatomic doping of N in the post-processing process (Oh et al., 2019). With the study of N doping, the content of N doping is a key factor to affect the performance of catalyst. For example, Huang et al. (2017) reported a facile one-step pyrolysis strategy to synthesize Co/N-carbon in argon atmosphere. As illustrated in Figure 3A, the overpotential of Co/N-carbon is 103 mV (vs. RHE) at 10 mA cm^−2^. The dramatic enhancement of catalytic activity was even more apparent of charge-transfer resistance (R_ct_) for the Co/N-carbon (45 Ω) (Figure 3B). Experimental results prove that after doping with N, HER performance of Co/N-carbon has been significantly improved. There is almost no HER polarization curve shift for Co/N-carbon after 2,000 cycles, demonstrating their superior cycling durability. To further investigate the effect of N doping, they prepared various catalysts with different N contents. According to the linear sweep voltammetry (LSV) curves, the catalyst of N with 30 mg dosage shows the best HER performance, which further illustrated that the less dosage of N atom can't provide enough rich electronic. However, excessive dopant will reduce the graphitic carbon, and degrade the performance of HER. Therefore, the content of N is an important factor affecting the HER performance.

Dual-heteroatom-doped has become a burgeoning research topic to further boost the HER performance of Co-MOFs derived catalysts. Experimental results revealed that co-doped can downshift the valence bands of carbon matrix and reduce HER overpotential (Zheng et al., 2014; Jiao et al., 2016). In order to improve the catalytic performance of N doping, the use of co-doped is generally required. In the co-doped system, the second doping element is one of key problems needs to be tackled. A simple and effective strategy to solve such a problem is using two doping elements which play different roles on the carbon matrix. N-doping can promote electrocatalytic performance by enhancing the adsorption strength of anionic groups (-OH), optimizing the Δ${\text{G}}_{{\text{H}}^{*}}$ and ΔG_H2O_. For S doping, the electronegativity is almost the same as carbon, mainly by changing the electron spin density to improve the performance of the carbon matrix. The enhanced electrocatalytic activity of catalysts was ascribed to the optimized water and hydrogen adsorption free energy by N, S atoms co-doping (Weng et al., 2018). Weng et al. (2018) demonstrated a facile preparation of S-doping CoWP nanoparticles embedded in S- and N-doped carbon matrix [S-CoWP@ (S, N)-C] and further proved the synergistic effects between S and N. In addition, doping B and N into the carbon matrix is another effective approach. When one carbon atom is adjacent to two different doping atoms which have the same electron-absorbing or electron-giving effects on the middle carbon atom, it will not be conducive to the polarization of the electron cloud of the middle carbon atom. On the contrary, if the heteroatoms on the one side of the carbon atom are pushing electrons on the other side of carbon, it will promote the polarization of the electron cloud of the middle carbon atom (Liu et al., 2018). Therefore, through the synergistic effect of B and N, the catalytic performance of materials can be improved (Liu et al., 2018).

To summarize, both BIFs and ZIFs are bridged by imidazolate. Their derivatives are naturally B-or/and N-doping materials. According to the above description, optimum N-doping content and co-doping play a significant role for boosting the performance of HER. However, to elucidate the synergistic effect of heteroatomic doping remains a challenge.

### Co-MOFs Derived Metal Selenide

Cobalt selenide has been attracted considerable attention in the field of water electrolysis due to its excellent performance, high stability and low cost (Liu Y. et al., 2014; Wang et al., 2015; Li K. et al., 2016; Ao et al., 2018; Wang X. et al., 2018a,b; Yi et al., 2020). The electrocatalysis activity of cobalt selenide is mainly attributed to the number of active sites. Moreover, the density of states (DOS) could also determine material properties. For electrocatalysts, the DOS near Fermi level is responsible for the adsorption strength of catalysts. Kong et al. (2013) has prepared a various of first-row transition metal dichalcogenides (ME_2_, M = Fe, Co, Ni; E =S, Se) as HER catalysts in acidic media. For all samples, CoSe_2_ exhibits a high HER performance and shows a small Tafel slope (42.175 mV dec^−1^), which may be related to its unique electronic structure.

**Figure 4:**
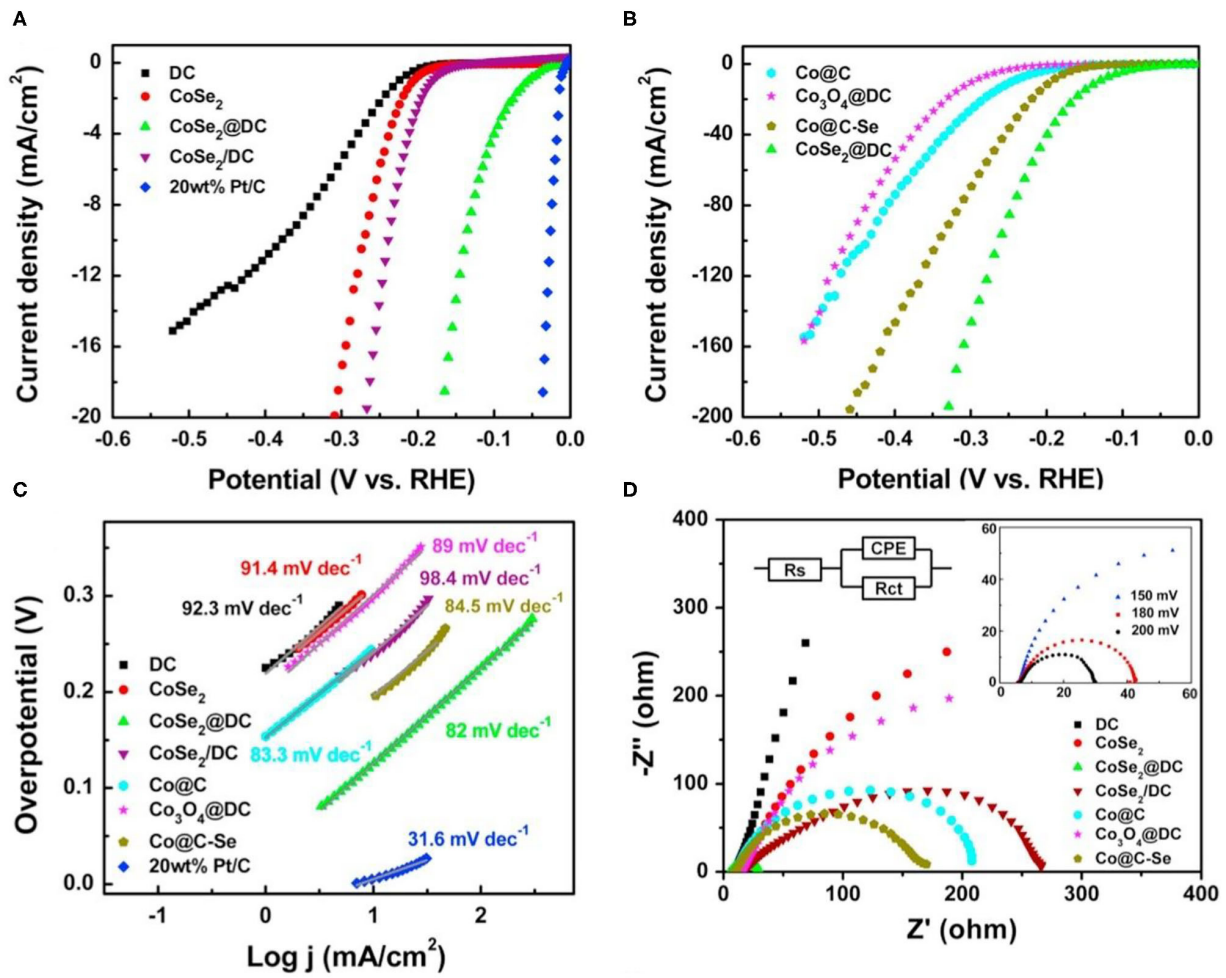
HER performances of Co-MOFs derived metal selenide (CoSe_2_@DC) in 0.5 M H_2_SO_4_. **(A,B)** Polarization curves of the different samples, **(C)** the Tafel plots from **(A,B,D)** Nyquist plots of the different samples. Reproduced with permission (Zhou W. et al., 2016). © 2016 Nano Energy. All rights reserved.

However, CoSe_2_ has fewer active sites due to easy agglomeration, which limits its application (Liu Y. et al., 2014; Kim et al., 2017; Wang F. et al., 2019; Wang X. et al., 2019; Ding et al., 2020). To overcome this drawback, assembling catalysts with conductive carbon, such as carbon fiber paper (Park and Kang, 2018), carbon nanotubes (Zhou W. et al., 2016; Park and Kang, 2018; Ding et al., 2020), has been demonstrated to be an effective approach. However, the uniform dispersion of nanoparticle catalysts on carbon matrix is still a great challenge. Thus, using MOFs as precursors to introduce carbon nanomaterials has become a popular research subject. According to the morphological relationship between the carbon material and CoSe_2_, it can be divided into coating (Zhou W. et al., 2016; Meng et al., 2017; Lu et al., 2019; Ding et al., 2020) and loading (Park and Kang, 2018). By coating with carbon materials, the agglomeration and corrosion of CoSe_2_ can be largely restricted (Zhang F. et al., 2019; Zhang L. et al., 2019; Ding et al., 2020). Zhou W. et al. (2016) prepared the core-shell structure of CoSe_2_@DC with CoSe_2_ as the core and embedded CoSe_2_ with defective carbon nanotubes by a carbonization-oxidation-sialylation strategies. Polarization curves of the materials are shown in Figures 4A,B. The overpotential of CoSe_2_@DC is 132 mV at 10 mA cm^−2^. Additionally, the Tafel slopes are drawn to study the HER kinetics of the products (Figure 4C). The CoSe_2_@DC exhibits a Tafel slope of 82 mV dec^−1^, which is lower than that of other materials. Nyquist plots of all catalysts are given in Figure 4D, obviously, the R_ct_ of CoSe_2_@DC is far smaller than the other catalysts. In addition, Ding et al. (2020) synthesized a CoSe_2_@N/C-CNT catalyst by self-assembling Co^2+^ ions in Adenine (Ade) which is the source of C and N. N-doping bamboo-like carbon nanotubes is also used to prevent the agglomeration and corrosion of catalyst. This method provides strong inspiration for design encased core-shell structure, which might eliminate the issue of the agglomeration. Loading the sample on a carbon substrate is another method to mitigate the agglomeration (Sun et al., 2016; Park and Kang, 2018). Sun et al. (2016) reported that Co^2+^ and organic ligand are repeatedly introduced on the carbon fiber paper. Then, Co-MOFs is formed through the heterosexual attraction between the positively charged Co^2+^ and the negatively charged -COOH group on the carbon fiber paper. Because of the high conductivity of carbon fiber paper, this material exhibits excellent electrocatalytic activity of HER. The time of introducing Co^2+^ and organic ligand was controlled in the synthesis process, which can avoid the agglomeration and produce the optimal loading catalyst with high performance.

### Co-MOFs Derived Metal Sulfide

Cobalt sulfides have got tremendous attention due to their intrinsic merits including low cost, easy synthesis and remarkable chemical bond between Co and S (Li K. et al., 2016; Li Z. Q. et al., 2016). The importance of S species in improving the HER performance of Co-based MOFs materials has been confirmed by Staszak-Jirkovsky et al. (2016). It has been known that the electronegativity of S is larger than that of Co (Liu et al., 2019b), the electron transfer from Co to S will increase the electron cloud density around S atoms (Liu S. et al., 2020a). The integrated effects of these factors in Co-MOFs derived metal sulfide ensure its excellent performances. Chen W. et al. (2019) fabricated the flower-like hybrid materials NCO@M (M = Co_3_O_4_, C, CoS, and CoSe) by ZIF-67 supported of Ni-Fe foam. From the SEM and TEM images, the morphology of NCO@CoS retains the flower-like structure while other samples are changed, suggesting that NCO@CoS has the strong chemical bond between Co and S elements. In addition, NCO@CoS also shows the excellent HER performances compared with the other three samples.

Cobalt sulfides have good corrosion resistance in alkaline solution, and the valence state of cobalt is abundant. Co and S can form various compounds, such as CoS, CoS_2_, Co_2_S_3_, Co_3_S_4_, Co_9_S_8_ (Chandrasekaran et al., 2019). The calcination time and atmosphere have been described as key conditions to determine the valence states of the cobalt. Sulfur could sublimate at high temperature, increasing the calcination time can reduce the sulfur content. When the experimental atmosphere is changed from Ar to H_2_/Ar, the S content will be further reduced. This might be ascribed to that S can react with H_2_ and thus reduce the content of S (Sun et al., 2018). However, most metal sulfide are semiconductors, when they are used as HER catalysts in acidic or alkaline solutions, insufficient conductivity and low stability seriously will degrade their electrocatalytic performance (Li H. et al., 2017; Li M. et al., 2017b). Incorporating carbon materials (e.g., carbon cloth and graphene oxide sheets) is a well-established strategy to enhance the conductivity of the electrocatalysts.

**Figure 5:**
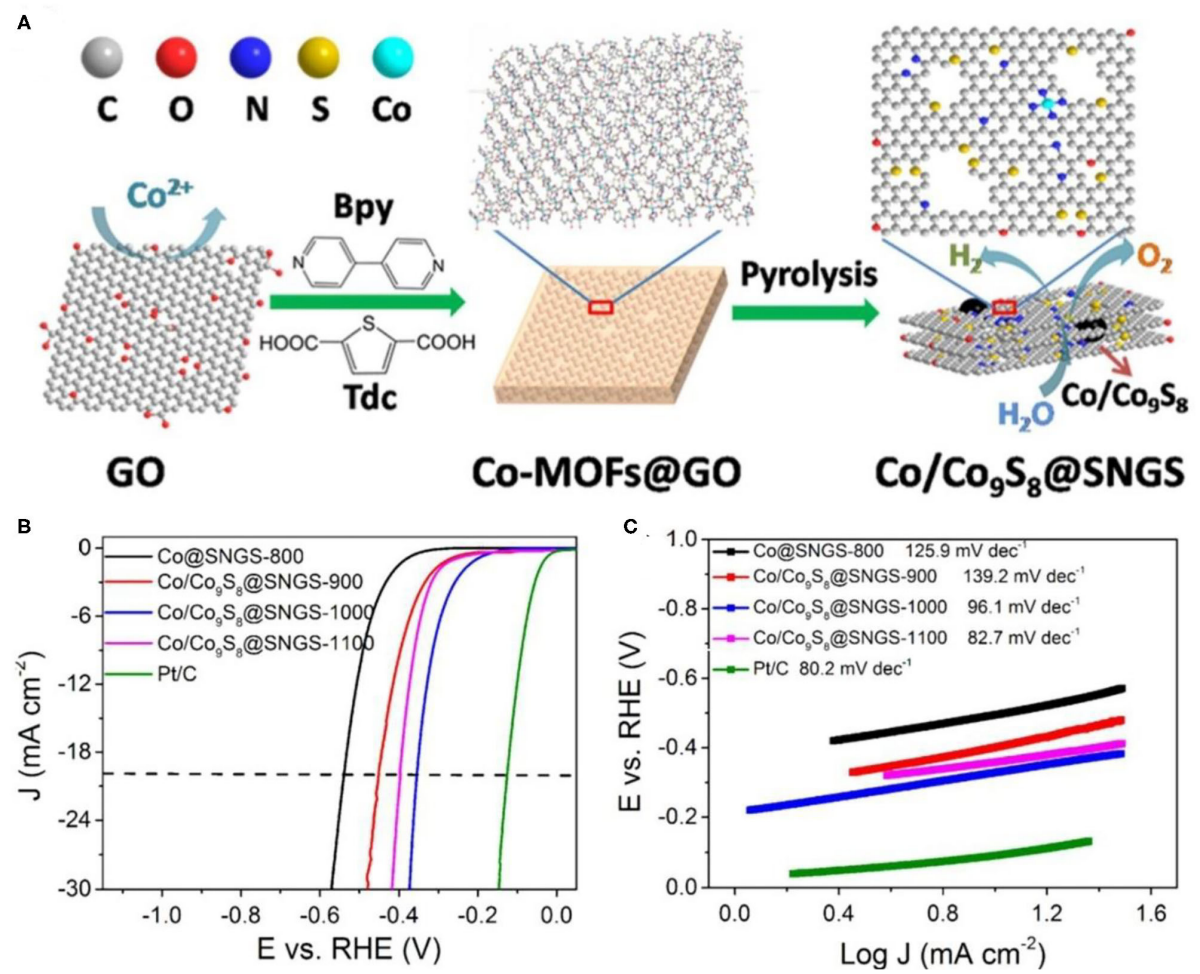
Co-MOFs derived metal sulfide for HER electrocatalysis under N_2_-saturated 0.1 M KOH solution. **(A)** A schematic illustration of the synthesis of Co/Co_9_S_8_@SNGS; **(B)** Linear sweep voltammetry curves of the different samples; **(C)** Tafel plots of the different samples. Reproduced with permission (Zhang X. et al., 2016). © 2016 Nano Energy. All rights reserved.

When the catalyst loading on the carbon matrix, the additional sulfur source will be partially doped in the carbon material (Wu et al., 2015). Since most organic ligands are rich in N, S, and N are usually co-doped in the carbon matrix (Li M. et al., 2017b; Wu et al., 2017b). Recently, S, N co-doping has stimulated intensive interest as an emerging method (Wang et al., 2017). Zhang X. et al. (2016) used S- and N-containing chemicals with fixed S/N atomic ratios as precursors to precisely control the doping of S and N in the carbon structure. As shown in Figure 5A, the Co/Co_9_S_8_@SNGS catalyst was synthesized by Co ions with S containing thiophene-2,5-dicarboxylate (Tdc) and N-containing 4,4′-bipyridine (Bpy) (Figure 5A). The two-dimensional network layer is formed by Tdc in the sample, and the connection between the two-dimensional network structure is realized by Bpy. The experimental results show that due to the periodic arrangement of the two connectors, the precise ratio of N and S can be achieved to 2.4:1. The electrocatalytic performance of the Co/Co_9_S_8_@SNGS-T (T = 900, 1,000, 1,100°C) and Co@SNGS-800 was evaluated in 0.1 M KOH solution. The onset potentials of the Co@SNGS-800, Co/Co_9_S_8_@SNGS-900, Co/Co_9_S_8_@SNGS-1000, Co/Co_9_S_8_@SNGS-1100, and Pt/C are 320, 250, 150, 240, and 0 mV (vs. RHE), respectively (Figure 5B). Additionally, Co/Co_9_S_8_@SNGS-T shows the smaller Tafel slope than that of Co@SNGS-800 (125.9 mV dec^−1^) (Figure 5C). The above HER results further confirmed that the interaction between Co and S can promote the performance of HER.

### Co-MOFs Derived Metal Phosphide

Recent years, transition metal phosphides (TMPs) have attracted wide attention due to their excellent HER activity, low cost and stability in acidic environments (Tabassum et al., 2017; Wang Q. et al., 2018; Wang X. et al., 2018a; Zhang et al., 2018; Ma X. C. et al., 2020). Unlike the typical layer-structured metal sulfides, TMPs tend to form more isotropic crystal structures. Due to the unique structure, metal phosphides usually exhibit abundant unsaturated coordination atoms on the surface. Therefore, TMPs are believed to have higher activity for HER than transition metal sulfides (Das and Nanda, 2016). P vapor is often used for TMP preparation, but it usually requires very high temperature (>500°C) due to the non-reactivity of P_4_ molecules (Zhang K. et al., 2017). While, such high temperature will cause the collapse of MOFs, thereby reducing the exposure of active sites and hindering electrons transport (Zhang L. et al., 2019). Hence, it is important to select an appropriate phosphorus source (Jia et al., 2017; Yang et al., 2017; Liu et al., 2019b). PH_3_ with high reactivity seems to be a better choice than P vapor, but it is extremely toxic, and has high risk during the experiment. In order to solve this problem, the use of NaH_2_PO_2_ as a phosphorus source can not only achieve low temperature phosphating (~300°C) but also ensure safety during the experiment (Liu Q. et al., 2014; Liu Y. et al., 2014; Zhang et al., 2015).

The low conductivity of cobalt phosphides, however, seriously limits their widespread application (Zhou D. et al., 2016; Zhou W. et al., 2016; Wu et al., 2017a; Zhang K. et al., 2017; Pan et al., 2018). Co-MOFs derived cobalt phosphides feature highly dispersed active phases in carbon matrix, which improving the conductivity of materials and making Co-MOFs derived cobalt phosphide an ideal catalyst for HER. In 2018, Hao et al. (2018) successfully embedded Co/CoP into a hairy N-doping carbon polyhedron (Co/CoP HNC). The N-doping carbon nanotube structure not only enhances the interface contact between catalyst and electrolyte, but also facilitates the charge transfer. Inspired by this structure, Pan et al. (2018) prepared the similar structure sample with core shell ZIF-8@ZIF-67 as the precursor to the CoP nanoparticles (NPs) into the hollow polyhedron N-doping carbon nanotubes (NCNHP). It is found that the CoP/NCHNP has high conductivity, which can be ascribed to the hollow polyhedron unique N-doping carbon nanotubes.

**Figure 6:**
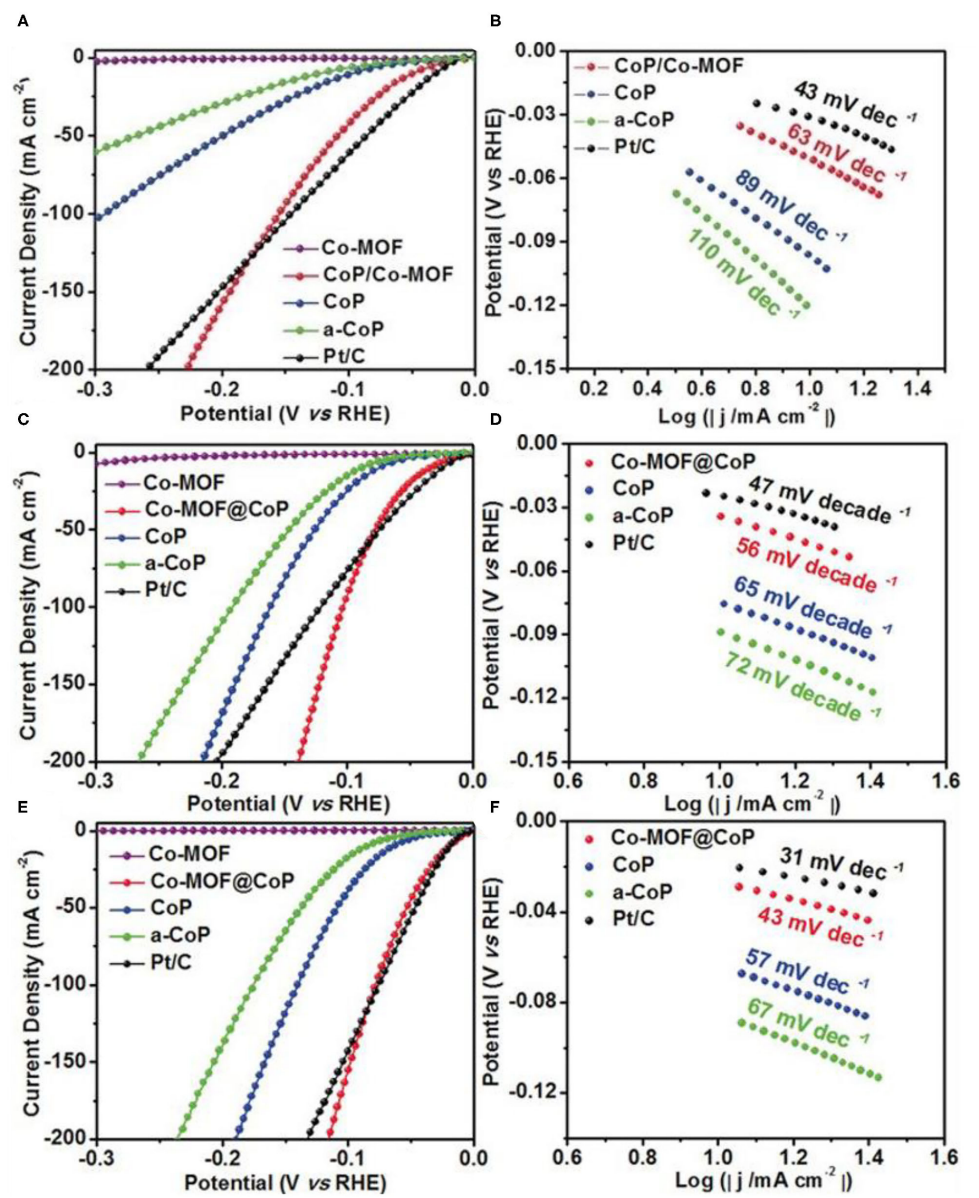
HER performances of Co-MOF derived metal phosphide. LSV curves **(A)**, Tafel slope **(B)** in 1 M PBS, LSV curves **(C)**, Tafel slope **(D)** in 1 M KOH and LSV curves **(E)**, Tafel slope **(F)** in 0.5 M H_2_SO_4_ of the different samples. Reproduced with permission (Liu et al., 2019a) © 2019 Angew. Chem. Int. Edit. All rights reserved.

The hybridization of Co and cobalt phosphides is another effective approach to enhance the conductivity (Masa et al., 2016; Xue et al., 2017). However, the higher concentration of P in the catalysts impedes the delocalization of cobalt atoms (Wang F. et al., 2019), which is a major reason why it has a low performance of HER. Hence, to control the concentration of P is still an urgent task. Liu et al. (2019a) proposed CoP/Co-MOF on a carbon fiber paper (CF) through a controllable partial phosphorization strategy. The optimized CoP/Co-MOF/CF exhibits outstanding HER performance in alkaline, acidic, and neutral conditions. Remarkably, the CoP/Co-MOF/CF achieves a current density of 10 mA cm^−2^ at an extremely low overpotential of 49, 34, and 21 mV in 1.0 M PBS, 1.0 M KOH, and 0.5 M H_2_SO_4_ solutions, respectively (Figures 6A,C,E). Figures 6B,D,F indicate that CoP/Co-MOF possesses the fast dynamics with the Tafel slope of 63, 56, and 43 mV dec^−1^ in 1.0 M PBS, 1.0 M KOH, and 0.5 M H_2_SO_4_ solutions, respectively. Both experiment and density functional theory (DFT) results show that the N atom in Co-MOF has large electronegativity, the electrons transfer from CoP to Co-MOF increases the positive charge of Co atoms. Positive charge Co atoms interact with the negative charge oxygen atoms in water is conducive to the adsorption and activation of water molecules, thus improve the performance of HER.

**Figure 7:**
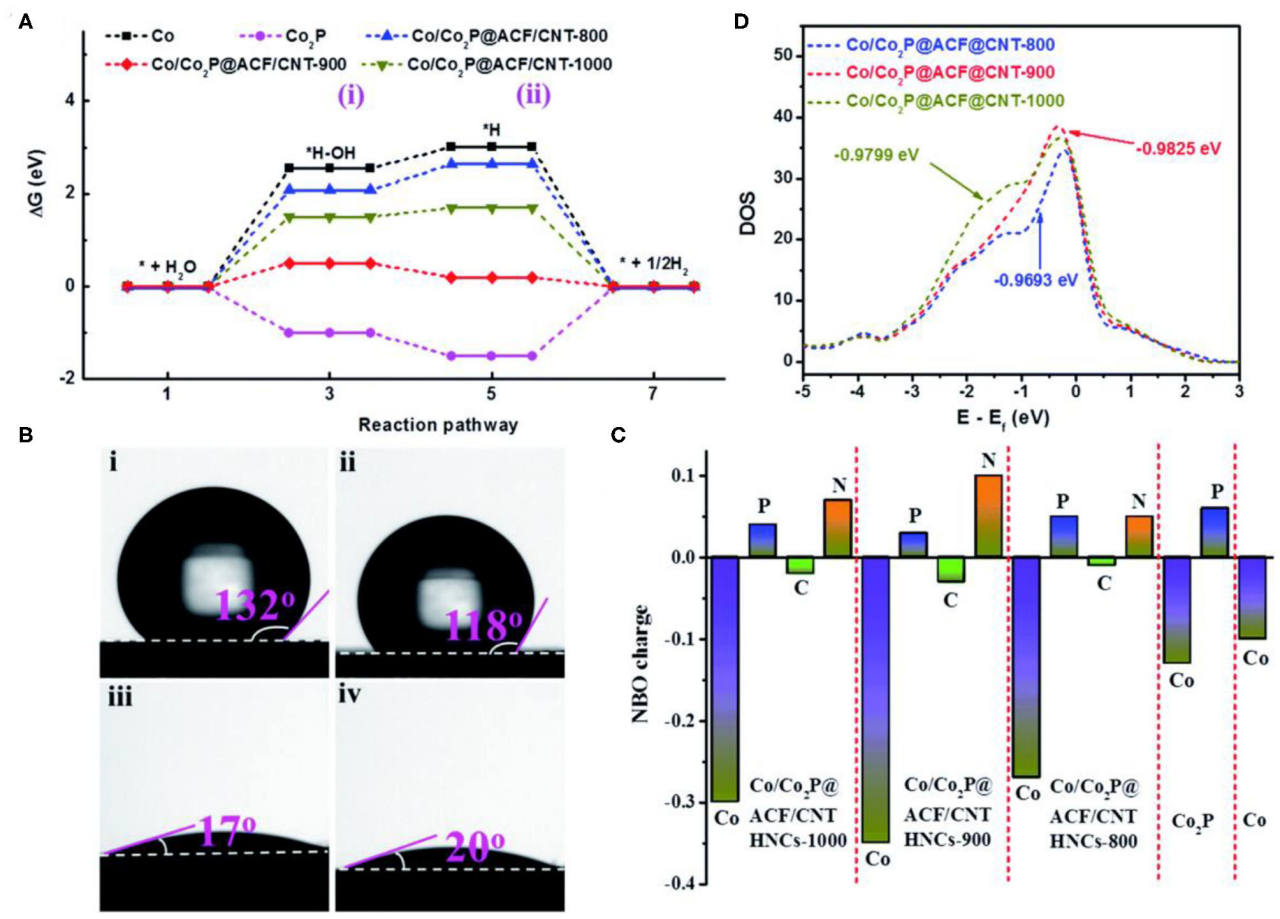
HER performances of Co-MOFs derived metal phosphide synthesized by controlling the pyrolysis temperature. **(A)** Calculated free energy diagram of the HER for (i) *H_2_O and (ii) *1/2H_2_ on the different samples, **(B)** The contact angles of a drop of 1.0 M KOH on (i): ZIF67 precursor, (ii): Co/Co_2_P@ACF/CNT HNCs-800, (iii): Co/Co_2_P@ACF/CNT HNCs-900, and (iv): Co/Co_2_P@ACF/CNT HNCs-1000, **(C)** Calculated DOS of the different samples, **(D)** NBO charge distribution. Reproduced with permission (Wang F. et al., 2019). © 2019 J. Mater. Chem. A. All rights reserved.

In addition to partial phosphorization strategy, annealing temperature is another method to control the doping content of P. High temperature annealing could accelerate the loss of phosphorus and increase the content of Co (Wang F. et al., 2019). Wang F. et al. (2019) prepared the component controllable Co/Co_2_P@ACF/CNT HNCs materials through simple etching-pyrolysis-phosphate process. From the free energy calculation results (Figure 7A), Co/Co_2_P@ACF/CNT-900 has an optimal adsorption energy for water activation. The water contact angle (Figure 7B) shows that Co/Co_2_P@ACF/CNT-900 has a lower H^*^ intermediate adsorption energy. This indicates that Co/Co_2_P@ACF/CNT-900 is conducive to the adsorption of water, and promotes the Volmer steps, further enhances the HER performance. In addition, the d-band center of Co/Co_2_P@ACF/CNT-900 is close to the Fermi level, which possess the moderate H adsorption energy (Figures 7C,D). The subsequent electrochemical test was consistent with the above calculations, Co/Co_2_P-@ACF/CNT HNCS-900 exhibits the best HER performance. What's more, the phosphating degree could be controlled by changing the mass ratio of phosphorus source and Co during the experiment. Xue et al. (2017) synthesized a novel Mott–Schottky Co/Co_2_P microspheres (Co/Co_2_P@C) catalyst through carbonization and gradual phosphorization of Co-based MOFs. The hybridization between cobalt and Co_2_P can form the Mott-Schottky effect, which could effectively promote the electron transfer.

### Synergies With Other Metals

In addition to Co ion, the presence of other metals in the nodes or anchoring other metal ions into MOFs pores can obtain mixed Co-metal MOFs (CoM-MOFs). Compared with single metal Co-MOFs, CoM-MOFs exhibits better HER performance due to the increased active sites (Singh et al., 2019) or optimized absorption and desorption of hydrogen intermediates (Yilmaz et al., 2017; Li D. et al., 2018; Li X. et al., 2018; Yu et al., 2018; Chen W. et al., 2019; Feng et al., 2019; Zhang L. et al., 2019). In order to further improve the performance of the CoM-MOFs, it is important to understand the reaction mechanism of this materials. Increasing the level of the d-band center of metal ions could enhance the interaction between metal and the adsorbed molecules (Hammer and Norskov, 1995; Skúlason et al., 2010; Zheng et al., 2014; Chen et al., 2018). Therefore, regulating the d band center of Co can adjust the interaction between catalysts and hydrogen intermediates, optimize the Δ${\text{G}}_{{\text{H}}^{*}}$ (Ahn et al., 2018). There are two strategies to modulate the d-band center of Co: (1) adopting two metals with different electronegativities to promote the electron transfer, and adjusting the level of the d-band center of Co (Xu et al., 2018; Lian et al., 2019; Qiao et al., 2020). (2) regulating the lattice parameters and bandwidth of Co, thereby changing the height of the d-band center of Co (Lai et al., 2019; Wang X. et al., 2020; Zhang et al., 2020).

**Figure 8:**
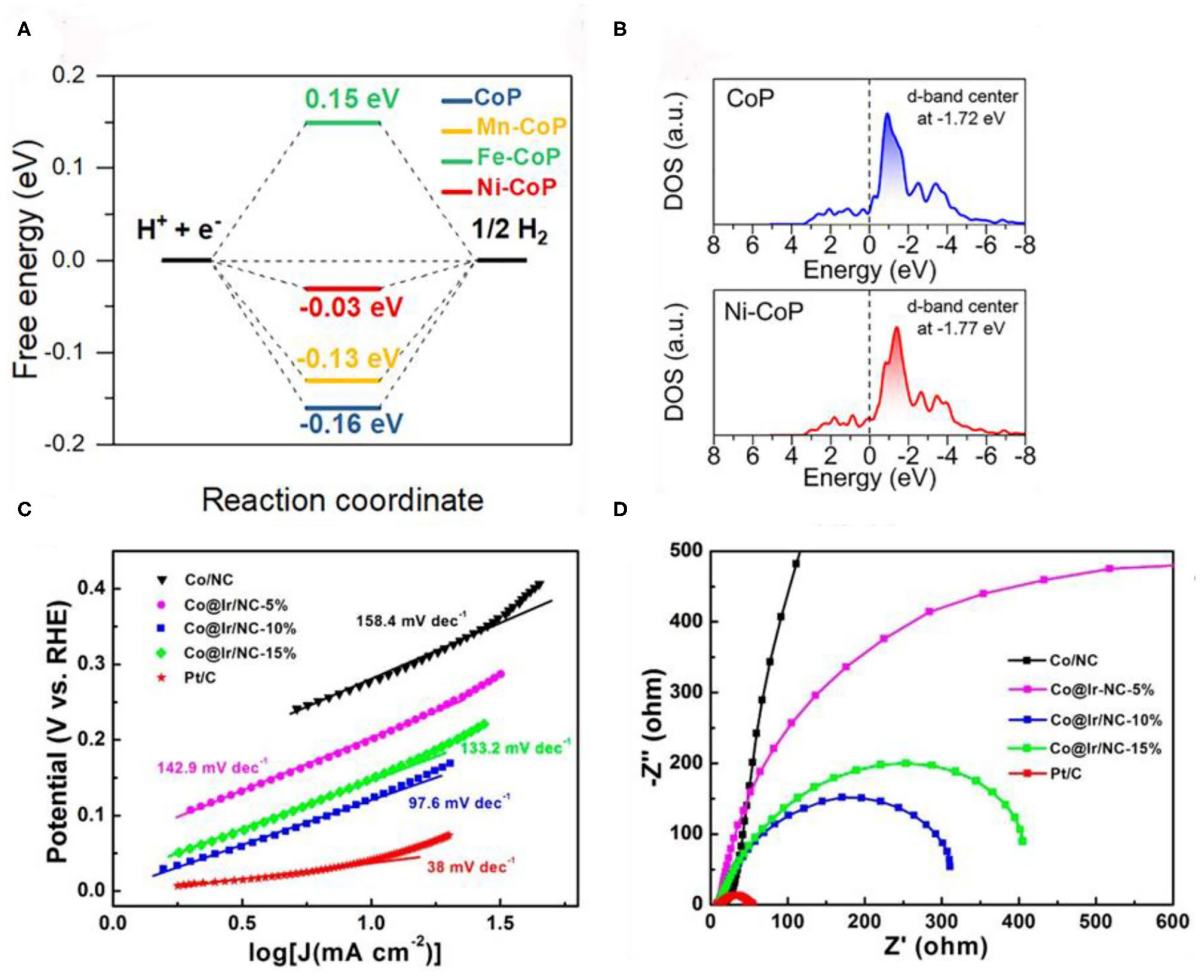
**(A)** The calculated free-energy diagram of the different samples, **(B)** Calculated DOS curves for CoP and Ni-CoP. Adapted with permission from Pan et al. (2019). © 2019 Nano Energy. All rights reserved. **(C)** Tafel plots of different catalysts in 1 M KOH solution. **(D)** Electrochemical impedance spectra of various catalysts at −0.07 V vs. RHE in 1 M KOH solution. Reproduced with permission (Li D. et al., 2018). © 2018 ACS Sustain. Chem. Eng. All rights reserved.

Recently, Pan et al. (2019) prepared M-doped CoP (M = Ni, Mn, Fe) on a hollow polyhedral framework (HPFs) by self-template transformation (STT) strategy. With Fe, Mn, and Ni doping, the doping ions substituted some Co^2+^ ions in CoP. DFT study (Figure 8A) suggested that Ni-doped CoP had the optimal hydrogen adsorption free energy. The doping of Ni atoms will lead to the transfer of electron from doping metal to Co atoms, and could improve the performance of HER. DOS calculation further proved how it changes the electronic structure of CoP. As shown in Figure 8B, the d-band center decreases as Ni doping in CoP, thus decrease the binding strength of H. These studies indicate that the downshift of d-band center reduces the adsorption of H and increase the desorption of H, which can improve the HER performance. Similarly, doping of other metals could also adjust the d-band center of Co. Li D. et al. (2018), Li X. et al. (2018) prepared Co@Ir/NC-*x* catalyst through a galvanic replacement reaction between IrCl_3_ and Co/NC. According to the X-ray photoelectron spectroscopy (XPS) analysis, the binding energy of the Co 2p electrons of Co@Ir/NC-10% (781.9 eV) is higher than that of Co/NC (780.0 eV). This indicates that the electron transferred from the Co core to the Ir shell, which can significantly optimize the electrocatalytic performance. According to Figure 8C, the Tafel slopes of Co/NC, Co@Ir/NC-5%, Co@Ir/NC-10%, Co@Ir/NC-15%, and Pt/C are measured to be 158.4, 142.9, 97.6, 133.2, and 38 mV dec^−1^, respectively. The Co@Ir/NC-10% also shows the lowest charge transfer resistance (Figure 8D). Unlike the above post-modification method, Chen W. et al. (2019) used two metal salts as metal sources and 1,4-benzenedicarboxylic acid (1,4-BDC) as a linker to synthesize Co-Fe-P nanotubes. The charge transfer from Fe to Co of Co-Fe-P catalyst achieved the desirable electronic configuration and boost the HER performance.

**Figure 9:**
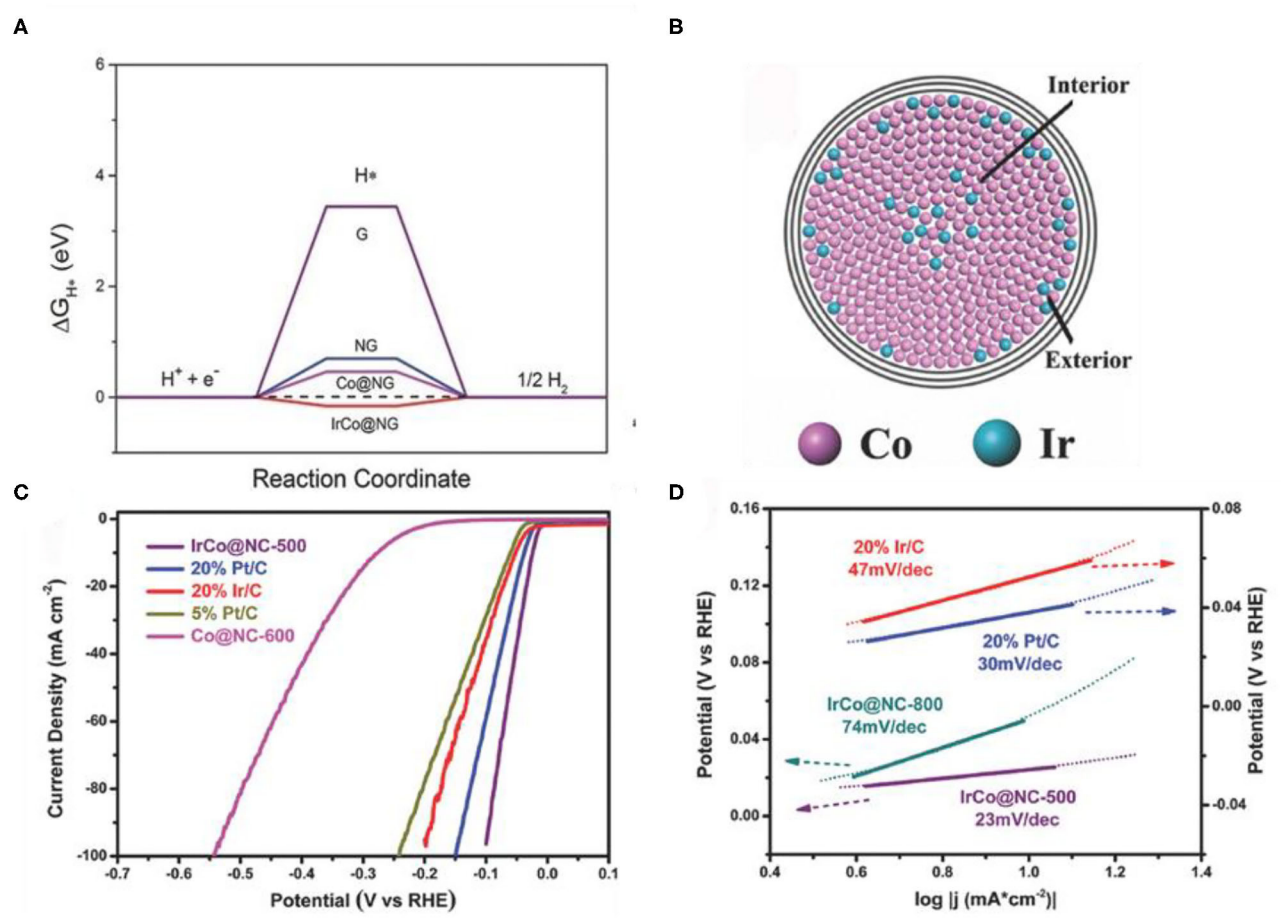
**(A)** Calculated free-energy diagram of HER at the equilibrium potential for different models, **(B)** Tentative model of an alloy core particle consisting of Co and Ir, **(C,D)** Electrocatalytic HER performance of the catalysts in N_2_-saturated 0.5 M H_2_SO_4_ solution. **(C)** Polarization curves of the different samples, **(D)** Tafel plots of the different catalysts. Reproduced with permission (Jiang et al., 2018). © 2018 Adv. Mater. All rights reserved.

Alloying is another simple and feasible strategy to adjust the d-band center (Wang X. et al., 2020). After alloying, the electronic structure of Co was altered, and hydrogen bonding energy was optimized, thus the HER performance was promoted (Greeley and Mavrikakis, 2004). The surface lattice strain and the coordination environment can be changed by adjusting the ratio of two kinds of metal atoms, thereby optimizing the d-band center of Co (Pan et al., 2019). Yang et al. (2015) prepared FeCo alloy nanoparticles by annealing of MOFs nanoparticles. Raising the annealing temperature, the average crystallite sizes of the crystal grain increases. After forming the alloy, the bond length of Fe-Co (2.18 Å) is lower than that of Co-Co, suggesting that the doping of Fe could result in strain effects. These results help to shift the d-band center of Co and increases the Δ${\text{G}}_{{\text{H}}^{*}}$. Jiang et al. (2018) prepared IrCo alloys (IrCo@NC) with a simple annealing strategy from Ir-doped Co-based MOFs. Compared with Co@NC, the d-band center of the IrCo@NC located in the vicinity of the Fermi level, which lead to the IrCo@NC has a moderate Δ${\text{G}}_{{\text{H}}^{*}}$ (Figure 9A). Due to the different atomic radius of Co and Ir, the lattice parameters of Co will be changed when Ir is introduced into the Co core (Figure 9B). From the electrochemical test, the IrCo@NC catalyst shows a low onset overpotential (24 mV) (Figure 9C) and a small Tafel slope (23 mV dec^−1^) (Figure 9D) than that of commercial Pt/C (30 mV dec^−1^).

CoM-MOFs is expected to provide a promising avenue in designing and developing novel catalysts, which can improve the performance by offering two different catalytic centers. Apart from doping other metals with different electronegativity, adjusting the lattice parameters of cobalt is another method to achieve the goals. However, how to control the distribution of metals in the catalyst is worth exploring.

## Conclusions and Perspectives

In summary, due to porous structures and variable chemical compositions, Co-MOFs have been proved to be an effective catalyst for HER. However, the HER performance of the original Co-based MOFs is still not very well. In this review, we summarize the recent efforts of Co-MOFs derived materials for HER: (1) Co-MOFs derivatives from various precursor; (2) Co-MOFs derived metal selenide; (3) Co-MOFs derived metal sulfide; (4) Co-MOFs derived metal phosphide; (5) synergies with other metals. To better understand the advantages of the above strategies, the HER performances of Co-MOFs derivatives catalysts are listed in Table 1. Those studies will provide some new insight in the development of Co-based catalysts for HER. Despite the impressive progress of Co-MOFs in this field, there are still many issues to be solved. In the end, to improve the catalytic performance of Co-MOFs derived materials for HER, the following urgent issues should be rationally considered.

1. It is important to explore various novel Co-based MOFs precursors. Currently, the preparation of Co-based MOFs is mainly come from ZIF-67 (Table 1), which possess chemical and thermal stability, and rich topological diversity. Nonetheless, exploring novel precursors to obtain more excellent Co-MOFs catalysts is of great significant for the development of hydrogen production in the future.
2. Optimizing the preparation condition of Co-based MOFs electrocatalysts is crucial. Although most MOFs are crystals, the conductivity of these materials are poorly or scarcely existing due to the insulating character of ligands. High temperature pyrolysis is necessary for MOFs precursor to improve the conductivity. However, the high temperature often leads to the aggregation of metal atoms and collapse of porous network of the MOFs-derived materials. Therefore, achieving an optimal balance among pyrolysis temperature, conductivity, metal particles distribution and surface structure of MOFs-based catalysts is still challenging.
3. The synergistic effect between Co and other metals can reduce the HER energy barrier and improve the catalysts performance. However, the existence of multiple metals increases the complexity of designing MOFs-based materials. The main challenge is how to control the synthesis of Co with other metals, including optimizing of the ratio of metal ions. In addition, the precise reaction mechanism of CoM-MOFs based catalysts is unclear. Hence, to obtain a high performance HER catalysts, it is necessary to have a fundamental understanding of the reaction mechanism for CoM-MOFs based materials.

**Table 1:** Summary of Co-MOFs derived electrocatalysts for HER.

	**Precursor**	**Loading amount [mg cm^**−2**^]**	**Electrolytes**	**Overpotential at 10 mA cm^**−2**^ [mV]**	**Tafel slope [mV dec^**−1**^]**	**References**
FeCo-600	ZIF-67	0.285	0.5 M H_2_SO_4_	262		Yang et al., 2015
Co/Co_2_P@C-10	ZIF-67	0.2	0.5 M H_2_SO_4_	192		Yu H. et al., 2020
			1.0 M KOH	158	56.35	
CoP@BCN	ZIF-67	0.4	0.5 M H_2_SO_4_	87	46	Tabassum et al., 2017
			1.0 M KOH	215	52	
			1M PBS	122	59	
Co-NC/CF	ZIF-67	0.649	1.0 M KOH	103	109	Huang et al., 2017
Co/NBC-900	BIF	2	1.0 M KOH	117	146	Liu et al., 2018
CoSe_2_/CF	ZIF-67	2.9	1.0 M KOH	52	95	Sun et al., 2016
S-CoWP@(S, N)-C	ZIF-67	0.75	1.0 M KOH	35	35	Weng et al., 2018
CoSe_2_@N/C-CNT	ZIF-67	0.255	0.5 M H_2_SO_4_	185	98	Ding et al., 2020
CoSe_2_@NC-NR/CNT	ZIF-67	1.3	0.5 M H_2_SO_4_		49.8	Park and Kang, 2018
CoSe_2_@DC	ZIF-67	0.357	0.5 M H_2_SO_4_	132	82	Zhou W. et al., 2016
CoSe_2_(400)-NC-800	ZIF-67	0.212	0.5 M H_2_SO_4_	234	95	Lu et al., 2019
NCO@CoS	ZIF-67	/	1.0 M KOH	100	68	Chen W. et al., 2019
Co_9_S_8_/CoS_1.097_/rGO	ZIF-67	1.684	0.5 M H_2_SO_4_	188	96	Sun et al., 2018
Co/Co9S8@SNGS-1000	ZIF-67	1	0.1 M KOH	350	96.1	Zhang X. et al., 2016
CoP/Co-MOF/CF	ZIF-67	5	0.5 M H_2_SO_4_	21	43	Liu et al., 2019a
			1.0 M KOH	34	56	
			1M PBS	49	63	
CoP–CNTs	ZIF-67	0.267	0.5 M H_2_SO_4_	139	52	Wu et al., 2017a
Co/CoP–HNC	ZIF-67	0.19	1.0 M KOH	180	105.6	Hao et al., 2018
CoP/NCNHP	ZIF-67	0.390	0.5 M H_2_SO_4_	140	53	Pan et al., 2018
		0.390	1.0 M KOH	115	66	
FexCo_2_-xP	ZIF-67	4	1.0 M KOH	114	97	Singh et al., 2019
Co@Ir/NC-10%	ZIF-67	0.202	0.5 M H_2_SO_4_	29.4	41.9	Li D. et al., 2018; Li X. et al., 2018
			1.0 M KOH	121	97.6	
NC@Cu-Co-W-C-700	ZIF-67	2	1.0 M KOH	98	50	Qiao et al., 2020
Co_0.6_Fe_0.4_P-1.125		0.270	0.5 M H_2_SO_4_	97		Lian et al., 2019
	ZIF-67		1.0 M KOH	133	61	
			1M PBS	140		
NiCoN/C	ZIF-67	/	1.0 M KOH	103		Lai et al., 2019
Co-NCF@600-Ni	ZIF-67	0.28	1.0 M KOH	157	112	Zhang et al., 2020
Ni-CoP/HPFs	ZIF-67	0.796	0.5 M H_2_SO_4_	144	52	Pan et al., 2019
			1.0 M KOH	92	71	
IrCo@NC-500	ZIF-67	0.285	0.5 M H_2_SO_4_	24	23	Jiang et al., 2018
N/Co-PCP//NRGO	ZIF-67	0.714	0.5 M H_2_SO_4_		126	Hou et al., 2015
Co-NC/CF	ZIF-67	1	1.0 M KOH	157	109	Huang H. et al., 2020b
Co/Co_9_S_8_	ZIF-67	0.64	1.0 M KOH	216	80	Du et al., 2019
MOF-CoSe_2_	ZIF-67	0.539	0.5 M H_2_SO_4_		42	Lin et al., 2017
CoP - NB	ZIF-67	0.707	0.5 M H_2_SO_4_		51	Wang X. et al., 2018a
CoPS@NPS-C (4 wt%)	ZIF-67	0.357	0.5 M H_2_SO_4_	93	63	Hu et al., 2018
Co_0.75_Fe_0.25_-NC	ZIF-67	0.212	1.0 M KOH	202	67.96	Feng et al., 2018
Zn_0.3_0Co_2.70_S_4_	ZIF-67	0.285	0.5 M H_2_SO_4_	80	47.5	Huang et al., 2016

## Author Contributions

YM prepared the outline of the review article and guided WH in preparing the first draft to final version. ML was responsible for the modification of language. JY put forward the central idea of the manuscript and gives final modification. All authors listed have made a substantial, direct and intellectual contribution to the work, and approved it for publication.

## Conflict of Interest

The authors declare that the research was conducted in the absence of any commercial or financial relationships that could be construed as a potential conflict of interest.
